# The Use of Myelinating Cultures as a Screen of Glycomolecules for CNS Repair

**DOI:** 10.3390/biology8030052

**Published:** 2019-06-28

**Authors:** George A. McCanney, Susan L. Lindsay, Michael A. McGrath, Hugh J. Willison, Claire Moss, Charles Bavington, Susan C. Barnett

**Affiliations:** 1 Institute of Infection, Immunity and Inflammation, College of Medical, Veterinary and Life Sciences, University of Glasgow, Glasgow G12 8TA, UK; 2 GlycoMar Limited, Malin House, European Marine Science Park, Dunbeg, Oban Argyll, Scotland PA37 1SZ, UK

**Keywords:** myelination, neurite outgrowth, cell culture, heparin sulphate, moderate throughput screen

## Abstract

In vitro cell-based assays have been fundamental in modern drug discovery and have led to the identification of novel therapeutics. We have developed complex mixed central nervous system (CNS) cultures, which recapitulate the normal process of myelination over time and allow the study of several parameters associated with CNS damage, both during development and after injury or disease. In particular, they have been used as a reliable screen to identify drug candidates that may promote (re)myelination and/or neurite outgrowth. Previously, using these cultures, we demonstrated that a panel of low sulphated heparin mimetics, with structures similar to heparan sulphates (HSs), can reduce astrogliosis, and promote myelination and neurite outgrowth. HSs reside in either the extracellular matrix or on the surface of cells and are thought to modulate cell signaling by both sequestering ligands, and acting as co-factors in the formation of ligand-receptor complexes. In this study, we have used these cultures as a screen to address the repair potential of numerous other commercially available sulphated glycomolecules, namely heparosans, ulvans, and fucoidans. These compounds are all known to have certain characteristics that mimic cellular glycosaminoglycans, similar to heparin mimetics. We show that the N-sulphated heparosans promoted myelination. However, O-sulphated heparosans did not affect myelination but promoted neurite outgrowth, indicating the importance of structure in HS function. Moreover, neither highly sulphated ulvans nor fucoidans had any effect on remyelination but CX-01, a low sulphated porcine intestinal heparin, promoted remyelination in vitro. These data illustrate the use of myelinating cultures as a screen and demonstrate the potential of heparin mimetics as CNS therapeutics.

## 1. Introduction

The repair of the central nervous system (CNS) is a complex process, which requires a multifactorial approach. Identifying molecules that promote CNS repair is an important aspect of developing therapies for regenerative medicine. We have devised a mixed neural cell culture that can be used to study developmental myelination, neurite outgrowth, and re/myelination after injury. The cultures are generated from dissociated embryonic rat spinal cords and develop over time to form many myelinated internodes separated by nodes of Ranvier [1]. They have allowed us to examine factors that regulate the process of myelination over time and we have, therefore, termed them myelinating cultures-development (MC-Dev). These cultures have been used to study the interaction between oligodendrocytes and axons during the process of myelination [2] and lead to the identification of the novel function of chemokines as regulators of myelination. Notably, C-X-C motif chemokine 10 [3] and connective tissue growth factor [4] can inhibit the development of myelination, while C-X-C motif chemokine 12 can promote myelination [5,6].

**Figure 1:**
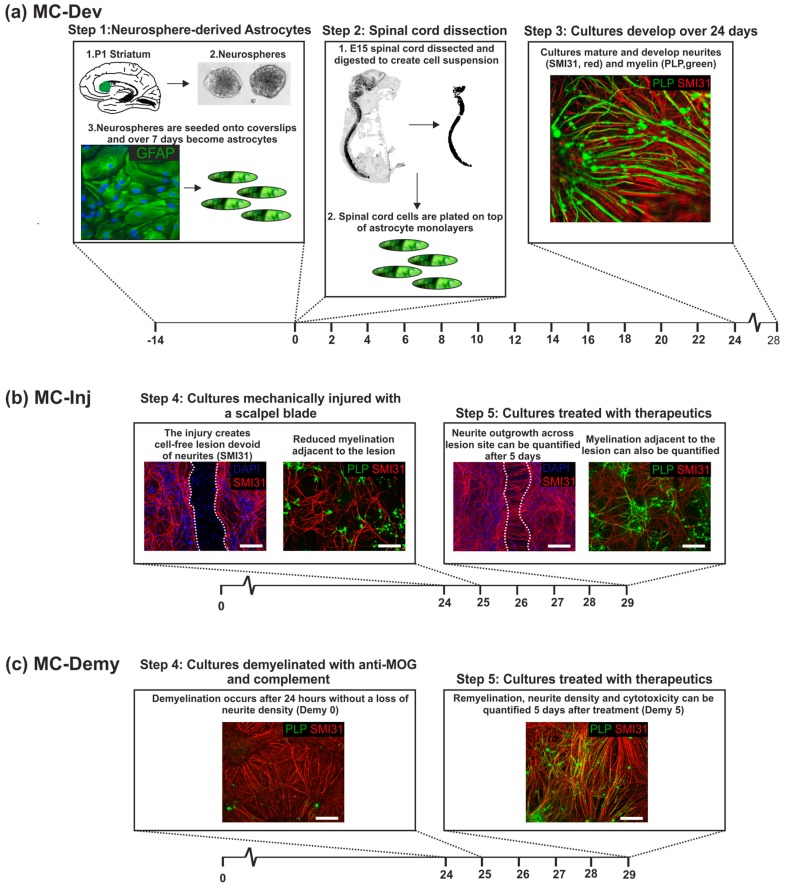
Schematic of in vitro myelinating cultures MC-Dev, MC-Inj, and MC-Demy. (**a**) MC-Dev are set up using astrocytes derived from neurospheres (Step 1, GFAP stains astrocytes in green) and mixed spinal cord cells derived from embryos at day 15 (Step 2) and allowed to develop for at least 24 days (Step 3). Therapeutics can be introduced from when the process of myelination begins (day 12–16) to determine their effect on de novo myelination. (**b**) The cultures can be used to mimic CNS injury (MC-Inj) by cutting with an 11 mm single edge razor blade pressed gently across the center of the coverslip on day 24 (Step 4). The day after injury, cultures can be treated with therapeutics and the extent of neurite outgrowth (SMI31 stains axons in red, DAPI stains nuclei blue) or myelination (PLP stains myelin in green) adjacent to the injury site quantified (Step 5). Dotted white line demarcates injury site. (**c**) To study remyelination, the cultures are demyelinated by overnight incubation with the Z2 antibody that recognizes myelin oligodendrocyte glycoprotein (MOG) and rabbit complement at 37 °C (Step 4). The day after demyelination the cultures can be treated with therapeutics and after 5 days the levels of remyelination quantified compared to non-treated control cultures (Step 5). Scale bars represents 100 μm.

We have further modified these cultures to study aspects of injury and disease. To generate an injury, we allowed the cultures to begin to myelinate and then made a lesion using a scalpel blade. Similar to a transection injury, we demonstrate how the neurites’ die back and the areas adjacent to the injury become demyelinated [7]. These cultures are termed myelinating cultures-injured (MC-Inj) and have been validated as a good model to study CNS damage using drugs known to induce repair, including Epac and Phosphodiesterase 4 (PDE4) inhibitors [7,8]. Lastly, we used these cultures to mimic diseases in which demyelination occurs, such as multiple sclerosis (MS). Axons were demyelinated using complement and the myelin oligodendrocyte glycoprotein (MOG) antibody, and the resulting cultures could be used to examine factors that promote remyelination [9,10]; we have termed these myelinating culture-demyelination (MC-Demy) (Figure 1). These cultures can be easily generated in large numbers, allowing us to screen several therapeutics simultaneously [1].

Previously, we demonstrated that low sulphated heparin mimetics (mHeps) could prevent features of in vitro astrogliosis [11,12,13]. mHeps are glycomolecules with similar structure to heparan sulphates (HSs), which reside either in the extracellular matrix (ECM) or on the surface of cells. mHeps have a repeating subunit of uronic acid linked to glucosamine, present as an alternating co-polymer of both iduronate- and glucuronate-containing sequences bearing N-sulphate, N-acetyl, and O-sulphate substitution. They can be used to mimic the activity of HSs, which are thought to modulate cell signaling by both sequestering ligands and acting as co-factors in the formation of ligand-receptor complexes [14]. Moreover, the biological activity of these molecules can be modified by its structure [14,15,16,17]. For this reason, we further investigated if these mHeps could influence other neural cells and promote CNS repair. We, therefore, applied the panel of mHeps with varying sulphation patterns to all three types of myelinating cultures. Although the mHeps had no effect on developmental myelination (MC-Dev), we found that low sulphated (LS) mHeps could promote myelination and neurite outgrowth and act via sequestering inhibitors of repair in MC-Demy and MC-Inj [10]. This led us to propose that LS-mHeps exert multiple beneficial effects on mechanisms supporting enhanced repair, and represent novel candidates as therapeutics for CNS damage.

These promising results have now led us to screen a further library of sulphated glycomolecules, composed of a panel of heparosans, ulvans, fucoidans, and a low sulphated heparinoid CX-01. Heparosan is a bacterial glycosaminoglycan (GAG) obtained as a K5 capsular polysaccharide from Escherichia coli with an analogous structure to hyaluronic acid [18]. The uronic acid epimerisation and various sulphate groups associated with heparan sulphate/heparin are absent from the K5 polysaccharide, along with the accompanying biological function [19]. However, the K5 polysaccharide can be enzymatically and chemically modified, producing a panel of sulphated and epimerised derivatives, which are suitable for exploring structure-activity relationships [20].

Marine organisms have become of interest to the pharmaceutical industry as a rich source of novel renewable therapeutic biologics, and fucoidans and ulvans are two such glycomolecules found in seaweeds [21,22,23]. Fucoidans are sulphated polysaccharides extracted from brown seaweed. The exact chemical composition varies between species, but predominantly, they are rich in galactose and sulphated fucose chains. Numerous beneficial neurological effects have been described, including protection from neuronal death in 1-methyl-4-phenyl-1,2,3,6-tetrahydropyridine (MPTP) Parkinson mice [24] and reducing myelin phagocytosis through the inhibition of scavenger receptors in MS [25]. Ulvans are complex branched polysaccharides found in the cell wall of green algae, made up of a repetitive disaccharide of 3-sulphated rhamnose and either xylose, iduronic acid, or glucuronic acid [26,27]. Ulvans have been suggested to display antioxidant and anti-tumour effects [28,29]. Based on such characteristics, and their unique structures, ulvans have been used in numerous biomaterials and bioscaffolds to aid repair and drug delivery [30,31]. Due to their wide-range of bio-activities, and their sulphated saccharide structures, fucoidans and ulvans were selected as potentially important therapeutic glycomolecules to screen using our in vitro CNS models.

The final compound in our library of glycomolecules was the low anticoagulant 2-O, 3-O desulphated heparin (ODSH, CX-01), which is a porcine intestinal heparin derivative that retains many heparin anti-inflammatory properties. It is well-characterised, commercially available, and is currently involved in a clinical trial for acute myeloid leukemia [32]. It was also used as an early therapeutic intervention after traumatic brain injury and showed beneficial functional outcomes [33]. We were, therefore, interested to determine if such beneficial effects could be observed in our in vitro CNS injury models.

Using our three complex neural cultures (MC-Inj, MC-Dev, and MC-Demy), we were able to screen this library of sulphated polysaccharides to establish any effect on re/myelination and neurite outgrowth. We have identified a number of interesting compounds, demonstrating the suitability of these complex cultures for screening potential therapeutics.

## 2. Materials and Methods

### 2.1. Library of Glycomolecules

Heparosans were gifted by Glycores 2000 Srl (Milan, Italy), and ulvans were produced and modified by Glycomar Ltd. (Oban, UK). The fucoidans were gifted for research use by Marinova Pty Ltd. (fucoidans 1, 3 and 5, Cambridge, Australia) and FMC Biopolymer (fucoidans 2 and 4, Vormedal, Norway). Glycomar Ltd. (UK) carried out desulphation and depolymerisation of fucoidans (fucoidans 1, 2 and 3). Cantex Pharmaceuticals (Weston, FL, USA) gifted the CX-01 compound.

### 2.2. Astrocytes Derived from Neurospheres

Neurospheres (NS) were generated from the striata of 1-day-old Sprague Dawley (SD) rat pups using a method modified by [34] and differentiated into astrocytes as described in [1,3]. Neurospheres were triturated and transferred to 13 mm poly-L-lysine (PLL; 13 µg/mL, Sigma, Gillingham, UK) coated coverslips (Corning, Deeside, UK) and incubated for a further 5–7 days in vitro (DIV) at 37 °C in an atmosphere of 7% CO_2_ until a confluent monolayer formed. Neurosphere-derived astrocytes were maintained in Dulbecco’s modified Eagle’s medium (DMEM-1 g/mL glucose, Life Technologies, Paisley, UK) with 10% fetal bovine serum (Sigma, UK) and 2 mM L-glutamine (Sigma, Gillingham, UK).

### 2.3. Myelinating Spinal Cord Cultures (MC-Dev)

Generation of rat myelinating cultures was based on previously described methods [1]. The spinal cord of E15.5 Sprague Dawley (SD) embryos were enzymatically dissociated and the cell suspension was plated at 150,000 cells per coverslip on top of the neurosphere derived astrocytes in a plating medium (PM) that contained 50% DMEM-1 g/mL glucose, 25% horse serum (Invitrogen), 25% HBSS (with Ca^2+^ and Mg^2+^, Life Technologies, UK), and 2 mM L-glutamine. Cells were left to adhere for 2 h at 37 °C, and then supplemented with 300 µL PM and 500 µL differentiation medium, which contained DMEM (4.5 g/mL glucose, Life Technologies, UK), 10 ng/mL biotin (Sigma, UK), 0.5% hormone mixture (1 mg/mL apotransferrin, 20 mM putrescine, 4 µM progesterone, 6 µM selenium (formulation based on N2 mix of [35] 50 nM hydrocortisone, and 0.5 mg/mL insulin known as DM+, or DM- if lacking insulin (all reagents from Sigma, UK). Each dish was fed three times a week with DM+ for 12 DIV then DM- for the following 16 DIV. To assess the effect of glycomolecules on developmental myelination, cultures were treated at 13 DIV and 20 DIV (1 nM), following which, the cultures were fixed and stained at 28 DIV.

### 2.4. Myelinating Injured Cultures (MC-Inj)

MC-Inj cultures were generated based on previously published methods [7,8]. At 24 DIV, when myelination had begun myelinating, cultures were cut using a 11 mm single edge razor blade (WPI, Aston, UK) pressed gently across the center of the coverslip. The cut created a focal cell free area (650 µm) with a decrease in adjacent neurite density and myelination levels, along with a very low number of neurites crossing the cut area site, referred to as the lesion. Using these cultures’ myelination, neurite density and outgrowth could be assessed using immunocytochemistry. The cultures were treated with the panel of glycomolecules at various concentrations at 25 DIV and allowed to recover for a further 5 DIV. Cultures were then fixed and stained as described in Section 2.6. 

### 2.5. Demyelinated Cultures (MC-Demy)

MC-Demy cultures were generated based on our previously published methods [9,10]. At 24 DIV, myelinating cultures were demyelinated by overnight incubation with the Z2 antibody (100 ng/mL Hybridoma, a kind gift from Christopher Linington, University of Glasgow), which recognises myelin oligodendrocyte glycoprotein (MOG) and a rabbit complement (100 μg/mL, Millipore, Watford, UK) at 37 °C. The demyelinated cultures were then treated with the panel of glycomolecules at various concentrations, following which, cultures were fixed and stained at 30 DIV. Additionally, a conditioned medium was collected at 28 DIV for CytoTox 96^®^ Non-Radioactive Cytotoxicity Assay (Promega G1780, Southampton, UK) to assess the viability of cultures under treatment following manufacturers guidelines.

### 2.6. Immunocytochemistry

Myelinating cultures were fixed with 4% paraformaldehyde (4% PFA, Sigma, UK) for 20 min at room temperature (RT) and permeabilised with 0.2% Triton X-100 (Sigma, UK) at RT for 15 min, once the washed cultures had been blocked with phosphate-buffered saline (PBS) with 0.2% porcine gelatin (blocking buffer, Sigma, UK) for 1 h at RT. The primary antibodies were diluted in a blocking buffer, and the cells were incubated for 1 h at RT. Mature myelin (proteolipid protein, PLP) was visualised using the AA3 antibody (1:100, anti-rat; hybridoma supernatant [36]), and a neurofilament was detected using SMI31 (mouse IgG1, 1:1500, BioLegend, London, UK). After washing, the cultures were incubated with the appropriate secondary antibodies for 45 min at RT, and mounted in a Vectashield (Vector Laboratories, Peterborough, UK).

### 2.7. Microscopy and Image Analysis

For all myelinating cultures, the coverslips were imaged using Olympus BX51 (Olympus, Essex, UK) or LAS AF Leica DM4000 B fluorescence microscopes (Leica Microsystems, Milton Keynes, UK), with the experimenter blinded to the conditions [6]. For quantitative analysis of MC-Inj, the region adjacent to the lesion edge running 0–670 µm across the length of the coverslip was imaged at 20× magnification to allow quantification of neurite density and myelination at the end of the experiment. For quantification of neurite outgrowth, the entire length of the injury site was imaged, with the exception of areas that were folded due to the mechanical effect of cutting, which were not imaged [7]. For MC-Dev and MC-Demy quantification of re/myelination and neurite density, images were taken at 10× magnification (as seen from example images in Figure 1). For each coverslip, 10 images were taken randomly, covering the entire coverslip. In each biological repeat, coverslips were stained in triplicate; thus, 30 images were taken per condition (at least n = 3 biological repeats). The analysis of treated cultures was carried out by a comparison to non-treated control cultures run in parallel. (non-treated injured cultures and non-treated demyelinated cultures for MC-Inj and Demy, respectively).

### 2.8. Re/myelination Quantification

For all myelinating cultures, the coverslips were stained using antibodies SMI31 and AA3 for neurites and myelin, respectively. Quantification of re/myelination was carried out using CellProfiler Image Analysis software (Broad Institute) and is available to download at https://github.com/muecs/cp [37,38]. CellProfiler uses pattern recognition software to distinguish between linear myelinated internodes and oligodendrocyte cell bodies. In this manner, we track the co-expression of myelin sheaths (PLP) and axons (SMI31), which ignores oligodendrocytes that do not contact axons, and therefore allows us to calculate the percentage of myelinated fibers. 

### 2.9. Neurite Outgrowth

Neurite outgrowth was defined as an SMI31 positive projection, which enters and crosses the lesion site. The number of neurites that crossed the lesion site was counted for each image. Any uninjured area around the lesion was excluded from analysis. Regions of neuronal bundles typically extended from one dense region and were, therefore, quantified as one projection, if individual neurites could not be detected. The number of neurites per image was averaged across the lesion and termed neurite outgrowth per field of view. 

### 2.10. Statistical Analysis

Data are presented as the mean + SEM and were analysed using ANOVA with a Dunnett post-test for multiple comparisons using Prism software version 6.0 (GraphPad Software, San Diego, CA, USA). Differences were considered significant at *p* < 0.05. Each experimental *n* was the average of 3 technical replicates. 

## 3. Results

### 3.1. Heparosans K5 NS and (Epi) K5 OS (H) Promote CNS Repair in MC-Inj

**Figure 2:**
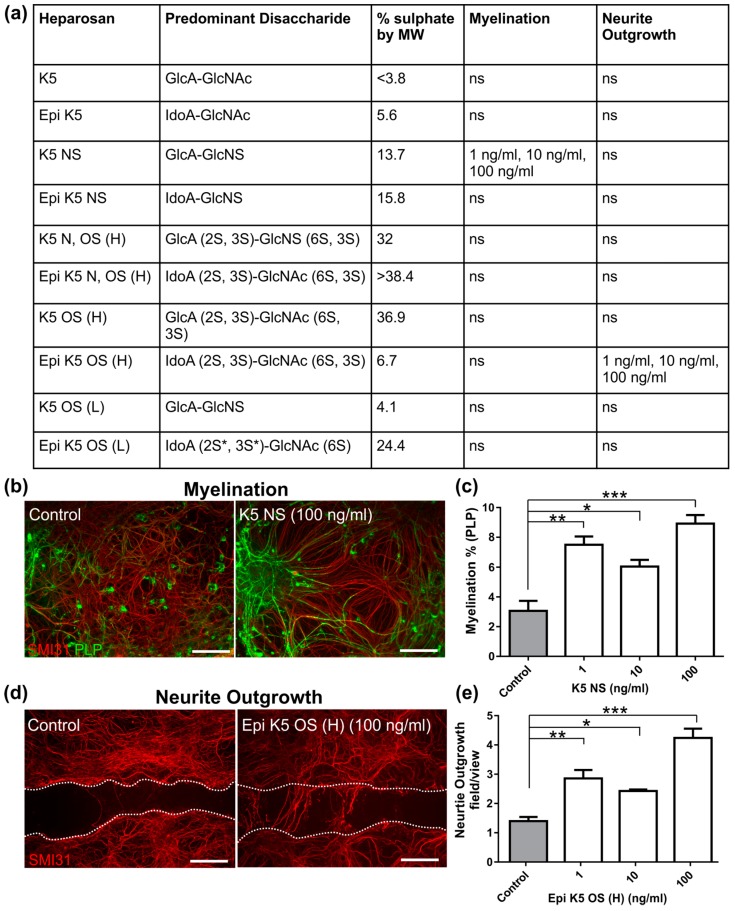
Heparosan screen using MC-Inj. (**a**) Table of the panel of heparosans used to treat MC-Inj detailing the predominant disaccharide, the % sulphate by MW (based on modified Terho method) and concentrations at which any significant increases were detected for each parameter tested. (**b**) Representative images of MC-Inj at 30 DIV showing areas adjacent to the lesion in control and after treatment with K5 NS at 100 ng/mL (SMI31 stains neurites in red, PLP stains myelin in green). (**c**) Quantification of K5 NS treatment shows that it significantly promotes myelination adjacent to the lesion at 1 ng/mL, 10 ng/mL, and 100 ng/mL compared to the non-treated injured control cultures (one-way ANOVA with Dunnett multiple comparison, ** *p* = 0.0015, * *p* = 0.015 *** *p* = 0.0003, respectively). (**d**) Representative images of MC-Inj lesion at 30 DIV showing the non-treated control and following treatment with Epi K5 OS at 100 ng/mL (SMI31 stains neurites in red). Injury site demarcated with white dotted lines. (**e**) Quantification of Epi K5 OS (H) treatment at 1 ng/mL, 10 ng/mL and 100 ng/mL showed significant increases in neurite outgrowth compared to the non-treated injured control cultures at all concentrations (one-way ANOVA with Dunnett multiple comparison, ** *p* = 0.0048, * *p* = 0.030 and *** *p* = 0.0001, respectively); ns, non-significant, Scale bars represent 50 μm in all images, error bars + SEM (all heparosan treatments n = 3; technical replicates n = 3).

Tables detailing the panel of heparosans used in this investigation are shown in Figure 2a. MC-Inj cultures were treated with a titration of each heparosan (1, 10, and 100 ng/mL) followed by an assessment of myelination and neurite outgrowth (summarised in Figure 2a). K5 NS at 1 ng/mL, 10 ng/mL, and 100 ng/mL was the only heparosan found to significantly promote myelination (~3 fold) compared to the non-treated control cultures (*p* = 0.0015, *p* = 0.015 and *p* = 0.0003, respectively; Figure 2b,c). However, its epimerised form did not have a similar pro-myelinating effect, suggesting that the conformation of the uronic acid residue is important for function. Interestingly, neither forms of this heparosans had any effect on neurite outgrowth. Conversely, O-sulphated heparosan Epi K5 OS (H) promoted neurite outgrowth compared to the non-treated control cultures (Figure 2d,e). Epi K5 OS (H) treatments at 1 ng/mL, 10 ng/mL, and 100 ng/mL resulted in a significantly increased neurite outgrowth of 51%, 43%, and 67%, respectively (*p* = 0.0048, *p* = 0.030 and *p* = 0.0001, respectively). These data imply that epimerisation is also a vital heparosan modification for the observed effect on neurite outgrowth. 

### 3.2. Heparosans Do Not Affect the Development of Myelination in MC-Dev or Remyelination in MC-Demy

**Figure 3:**
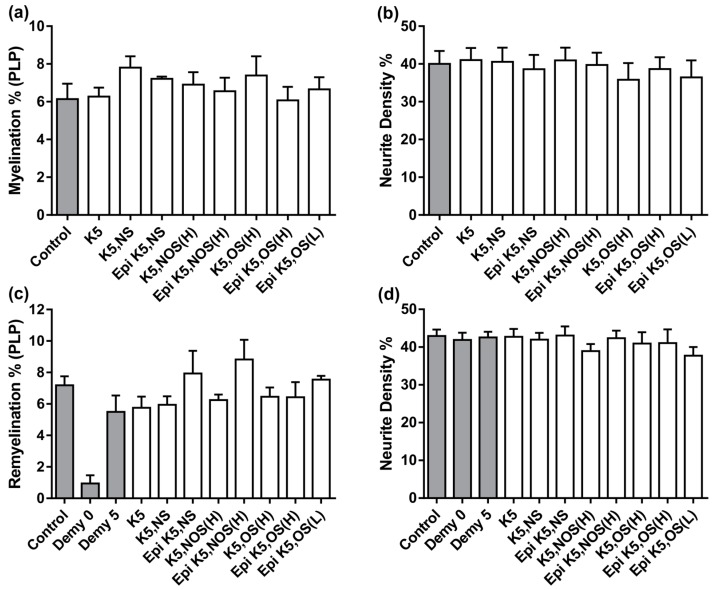
Heparosan screen using MC-Dev and MC-Demy. (**a**) MC-Dev were treated with each heparosan at 1 nM on 13 DIV and 20 DIV and allowed to mature until 24 DIV, at which point they were immunolabelled with SMI31 and PLP. There were no significant differences in the level of myelination found after treatment with any heparosan. (**b**) Quantification of neurite density also demonstrated no effect following treatment with any heparosan. (**c**) MC-Demy were treated with each heparosan at 1 nM on 25 DIV following overnight incubation with anti-MOG and rabbit complement. Quantification of remyelination showed no significant effect following treatment with any heparosan. There was a trend for increased remyelination following Epi K5 NS and Epi K5, NOS (H) treatments compared to the non-treated controls (Demy 5). However, this did not reach statistical significance. (**d**) The quantification of neurite density again showed no change after treatment indicating no adverse toxic effects of any heparosan on the cultures. Error bars + SEM (one-way ANOVA with Dunnett multiple comparison n = 4; technical replicates = 3).

The same panel of heparosans (detailed in Figure 2a) was used to treat MC-Dev and were found to have no effect on developmental myelination or on neurite density, similar to results previously published by us using sulphated polysaccharides [10] (Figure 3a,b). This suggests that the beneficial effect on myelination following K5 NS treatment in the MC-Inj screen is unlikely to be a direct effect on the compounds ability to promote oligodendrocyte precursor cell (OPC) differentiation and/or the process of oligodendrocyte ensheathment of axons. To determine whether pro-myelinating effects could be observed in a different injury model, we tested the panel of heparosans using MC-Demy (Figure 3c,d). Cultures were treated with each compound at 1 nM to enable a direct comparison between compounds. However, at this concentration, there were no significant improvements in remyelination observed for any of the panel, with only a small trend towards an increase after Epi K5 NS and Epi K5 NOS (H) treatment (Figure 3c,d). A dose response for Epi K5 NS was carried out and demonstrated no significant effect on remyelination [39]. 

### 3.3. Fucoidans or Ulvans Did Not Affect the Level of Remyelination in MC-Demy

**Figure 4:**
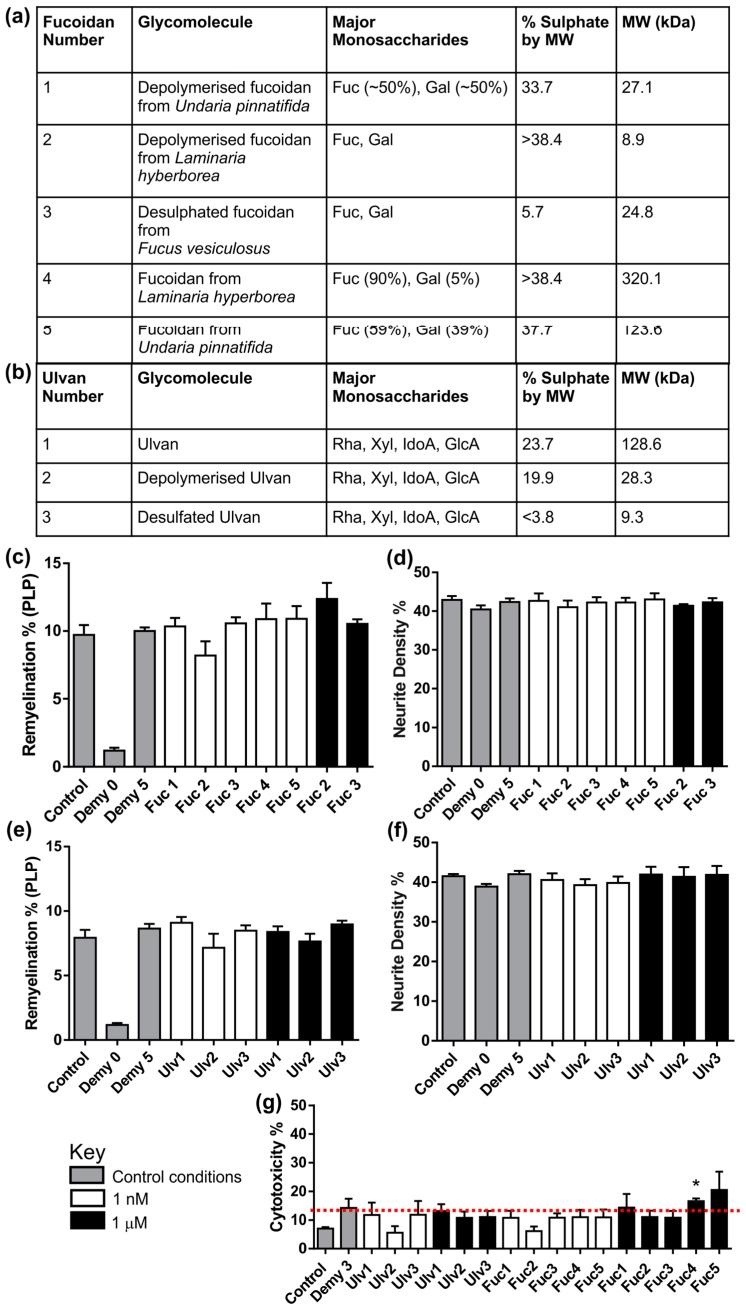
Fucoidan and Ulvan panel screen using MC-Demy. (**a**) Tables displaying the major monosaccharides, the % sulphate by MW (based on modified Terho method) and estimated MW determined by HPLC-SEC using dextran standards of the panel of Fucoidans (Fuc) and (**b**) Ulvans (Ulv) used to treat MC-Demy. (**c**) MC-Demy were treated with fucoidans at either 1 nM or 1 μM on 25 DIV and allowed to recover until 30 DIV, when they were immunolabelled with SMI31 and PLP. There was no significant effect on the level of remyelination after fucoidan treatment at either concentration tested. Fucoidan 1, 4 and 5 treatments at 1 μM were toxic and killed the cultures by 30 DIV, meaning that myelination and neurite density could not be quantified. (**d**) All fucoidans tested had no effect on neurite density at either concentration tested. (**e**) Quantification of remyelination following treatment with ulvans at either 1 nM or 1 µM suggests that none of these compounds had any effect compared to non-treated control cultures (Demy 5). (**f**) Quantification of neurite density showed no difference after treatment. (**g**) Conditioned media from the cultures were collected at 28 DIV, 2 days prior to immunolabelling and used to assess cytotoxicity based on LDH levels. The toxicity of Fucoidan 1, 4 and 5 was indicated in the early cytotoxicity assay with Fuc 4 already showing significant toxicity prior to culture death (one-way ANOVA with Dunnett multiple comparison, * *p* = 0.0366). Error bars + SEM (n = 4; technical replicates = 3).

Details of the panel of fucoidans and ulvans screened in MC-Demy are shown in Figure 4a,b. Fucoidans (Figure 4c,d) and ulvans (Figure 4e,f) were used to treat MC-Demy at 1 nM and 1 μM concentrations. None of the fucoidans or ulvans at either concentration had any effect on remyelination or neurite density. A toxicity screen was also carried out for each fucoidan and ulvan via collection of the growth medium from the cultures after treatment. This conditioned medium was assessed for cellular toxicity using a cytotox non-radioactive assay. It was found that treatments at the higher concentration of 1 µM for fucoidans 1, 4, and 5 (Fuc1, Fuc4, Fuc5) were toxic to cultures as indicated in the 28 DIV toxicity screen (Figure 4g). Conversely, none of the ulvans had any toxic effects even at 1 μM.

### 3.4. CX-01 Promotes Remyelination in MC-Demy

**Figure 5:**
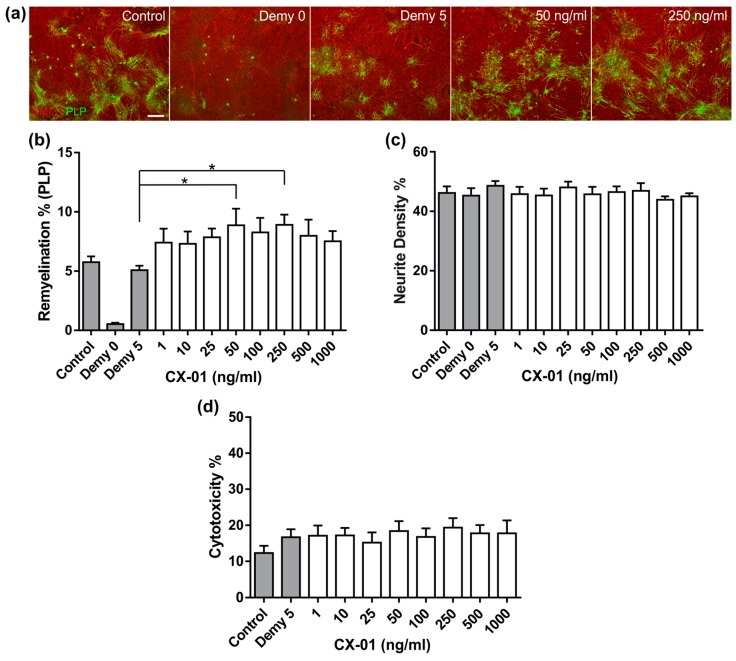
CX-01 promotes remyelination in MC-Demy. (**a**) MC-Demy was treated with CX-01 at 25 DIV, followed by immunolabelling with SMI31 (red, neurites) and PLP (green, myelin) 5 days later. Representative images of control cultures or those treated with anti-MOG and complement on either 25 DIV (Demy 0), or 30 DIV (Demy 5) and following CX-01 treatment at 50 ng/mL and 250 ng/mL. (**b**) Quantification showing treatment with CX-01 resulted in an average 75% increase in remyelination compared to the non-treated demyelinated control cultures on day 5 (Demy 5) at 50 ng/mL and 250 ng/mL (one-way ANOVA with Dunnett multiple comparison, * *p* = 0.0238 and 0.0222). (**c**) Quantification of neurite density showed no effect. (**d**) Conditioned media from the cultures were collected at 28 DIV, 2 days prior to immunolabelling, and used to assess cytotoxicity based on LDH levels. Quantification of cytotoxicity showed no change after treatment implying no adverse toxic effects on the cultures. Scale bars represent 100 μm in all images, error bars + SEM (n = 6; technical replicates = 3).

We also assessed the effect of CX-01 in MC-Demy using a titration ranging from 1 ng/mL to 1000 ng/mL. Treatment with CX-01 resulted in a significant increase in remyelination compared to the non-treated demyelinated control cultures (Demy5) at 50 ng/mL and 250 ng/mL (Figure 5a,b, *p* = 0.02). CX-01 had no effect on neurite density (Figure 5c). We also assessed if CX-01 was cytotoxic, by testing the growth medium after drug treatment. CX-01 was found not to be cytotoxic to the cultures supporting its potential use in treating neural cells (Figure 5d).

## 4. Discussion

In this study, we have used several modifications of myelinating cultures to screen a range of glycomolecules that mimic the HS found around the ECM and cell surface. HS are known to modulate the biological activity of a diverse range of molecules including chemokines, growth factors, morphogens, and adhesion molecules leading to a regulatory role in many physiological and pathological conditions [17]. They have also been used as pharmacological agents to treat a range of diseases [40,41]. The application of therapeutics for cancer treatment has been reported for non-coagulant heparin (SST001) and a phosphomannopentaose sulphate (PI-88) that targets heparanase [40,41]. PI-88 is in clinical trial for the treatment of liver cancer, non-small cell lung cancer, melanoma, and prostate cancer (ClinicalTrials.gov). Similarly, CX-01 is currently in clinical trials for myeloid leukaemia and cervical cancer [42]. Thus, it is clear that these HS mimetics have the potential to treat human disease and have been shown to produce very few side effects. However, there is no evidence from clinical trials that these molecules can be used to aid repair following CNS disease/injury.

Previously, we demonstrated using a panel of mHeps that the level of sulphation effected their potential to promote re/myelination and neurite outgrowth as well as decreasing characteristics of astrogliosis with only low sulphated mHeps promoting repair [11,12,13]. Moreover, their mechanism-of-action appeared to function via sequestering molecules that inhibited the repair process [10]. This was substantiated by other studies, which demonstrated that heparin promotes PNS myelination in DRG via the specific inhibition of the soluble immunoglobulin (Ig)-containing isoforms of neuregulin 1 (i.e., NRG1 types I and II), which negatively regulate myelination [43]. 

These data were encouraging and lead us to consider whether other similar sulphated glycomolecules would be useful for CNS repair. However, to test these reagents in animal models is both time consuming and requires a large number of animals. For this reason, we screened the panel of glycomolecules using our three modified myelinating cultures. It was found that only CX-01 and K5 NS demonstrated comparable pro-myelinating results, as seen previously for the low sulphated mHeps [10]. This suggests that N-sulphation is an important facet for promoting CNS myelination following injury. This is supported by a recent structure relationship activity study, which highlighted the importance of N-sulphation in facilitating specific protein interactions [16]. Conversely, the number of neurites crossing the lesion was only increased following treatment with highly sulphated epimerized Epi K5 OS (H), implying that O–sulphation might be a desirable feature for eliciting beneficial neurite outgrowth effects. The presence of O-sulphate moieties was deemed essential to the activity of K5 derivatives as FGF signalling inhibitors [44]. Furthermore, modulation of 6-O-sulphation has been shown to be imperative to Wnt signaling, which is known to regulate numerous aspects of neural precursor development [45,46,47,48]. Our data suggests that the epimerization state of the uronic acid may be a key structural feature for effecting myelination and neurite outgrowth. Thus, the epimerisation of glucuronic acid to its C5 epimer could be important for protein binding. The iduronic acid residue has additional conformational flexibility due to its ability to reside in the skew boat conformation; the subsequent kinking of the backbone could improve protein binding [49,50]. Interestingly, neither ulvans nor fucoidans had any effect on remyelination; these glycomolecules are composed of fucose/galactose and rhamnose/xylose/uronic acid respectively. This suggests that the glucosamine–uronic acid repeating sugar backbone present in K5 NS and CX-01 is imperative to the HS function in regulating CNS cell biology. The molecular weight and the concomitant degree of polymerisation is also a key feature for GAG activity—for example, the depolymerisation of fucosylated glycosaminoglycan reduced anticoagulant activity in vitro [51]. Hence, we assessed both the high and low molecular weight derivatives of fucoidans and ulvans using our cultures, but this had no effect on the lack of activity following treatment.

Using these modified myelinating cultures allowed us to pre-screen panels of glycomolecules, and to assess parameters, including myelination, remyelination, neurite density, and neurite outgrowth, before validation in vivo. Moreover, molecular techniques, such as PCR and Western Blotting, can be used to quantify data and examine the mechanism of action of treatment [7,8,10] or direct visualisation using time lapse [2]. Since these cultures contain many neural cells types, we can also make indirect observations by comparing drug treatment on the monocultures of OPCs, astrocytes, and microglia with the myelinating cultures. This, in turn, allows the investigation of cross-talk between the neural/immune cells present in the complex cultures, as previously discussed [52]. These cultures can, therefore, mimic aspects of the cellular interactions that occur in vivo.

## 5. Conclusions

In summary, the present study has demonstrated the efficacy of complex myelinating cultures modified to examine features of CNS repair to gain important information on a large panel of glycomolecules. Thus, we propose using such cultures as a useful, moderate screen to be used before testing potential therapeutic effects in animal models.
